# Genetics of Common Polygenic Ischaemic Stroke: Current Understanding and Future Challenges

**DOI:** 10.4061/2011/179061

**Published:** 2011-08-03

**Authors:** Steve Bevan, Hugh S. Markus

**Affiliations:** Stroke and Dementia Research Centre, St. George's, University of London, Cranmer Terrace, Tooting, London SW17 0RE, UK

## Abstract

Stroke is the third commonest cause of death and the major cause of adult neurological disability worldwide. While much is known about conventional risk factors such as hypertension, diabetes and incidence of smoking, these environmental factors only account for a proportion of stroke risk. Up to 50% of stroke risk can be attributed to genetic risk factors, although to date no single risk allele has been convincingly identified as contributing to this risk. Advances in the field of genetics, most notably genome wide association studies (GWAS), have revealed genetic risks in other cardiovascular disease and these techniques are now being applied to ischaemic stroke. This paper covers previous genetic studies in stroke including candidate gene studies, discusses the genome wide association approach, and future techniques such as next generation sequencing and the post-GWAS era. The review also considers the overlap from other cardiovascular diseases and whether findings from these may also be informative in ischaemic stroke.

## 1. Evidence for Genetic Factors in Stroke Risk

Stroke is the third commonest cause of death and the major cause of adult neurological disability, affecting both the developed world and increasingly having an impact in the developing world as well. It is also a major cause of dementia and the commonest cause of late onset epilepsy. Therefore, increasing our understanding of the risks, causes, and treatment of ischaemic stroke is of great importance. 

Stroke is itself a syndrome cause by a number of different disease processes. About 80% of strokes are ischemic and 20% are due to primary hemorrhage. In this paper we will only address the genetics of ischaemic stroke. While much is known about conventional risk factors such as hypertension, diabetes, and incidence of smoking, studies suggest these only account for a proportion of ischaemic stroke risk. Considerable evidence suggests genetic predisposition may explain some of the remaining risk, including evidence from both twin and family studies [1]. Family studies have shown differential association with different subtypes of stroke, suggesting these may have different underlying genetic risk factors [2, 3]. 

Further evidence for a genetic contribution to ischaemic stroke risk comes from animal models [4] and from the study of intermediate phenotypes such as carotid artery intima-media thickness (IMT) as a marker for large artery disease and MRI white matter hyperintensities as a marker for small vessel stroke. Twin and family history studies have shown these both have significant heritability (the proportion of stroke risk attributable to genetic risk factors) with estimates ranging from 55–71% for IMT [5–7] and 30–68% for WMH [8–10]. The identification of genetic variants predisposing to known stroke risk factors such as atrial fibrillation (AF) [11] and myocardial infarction (MI) and coronary artery disease (CAD) [12] further highlights the role of genetic predisposition in stroke risk. 

 The clearest evidence that genetics can cause ischaemic stroke comes from monogenic forms of the disease, although these account for only a relatively small percentage of overall ischaemic stroke incidence [13] and appear to have limited relevance to common polygenic stroke. As such they will not be considered as part of this paper in detail, but are covered in reviews elsewhere [14]. Therefore, considerable evidence suggests genetic factors do play an important role in ischaemic stroke, so why have so few genes been identified that contribute to this risk and why have other fields, including related cardiovascular disease phenotypes, been more successful?

## 2. Identification of Genetic Risk: Candidate Gene and Familial Linkage Studies

Until recently, identification of genetic variants contributing to disease has been attempted by 2 main techniques—candidate gene studies and familial linkage studies (See Box 1 for details of the different types of genetic investigation and their use). Of these, the candidate gene study has been the mainstay of genetic investigation into the vast majority of polygenic diseases thought to have a genetic component. Typically, a gene identified as a “candidate” is hypothesised to be involved in stroke risk, and then, genetic variants, usually single nucleotide polymorphisms (SNPs), are identified within that gene. The frequency of the SNPs is then determined in a series of cases and controls and the two compared. 

 The vast majority of candidate gene studies in ischaemic stroke have turned out to be disappointing. Reasons for this include insufficient sample size, a failure to replicate results initially reported as significant, poor stroke subtyping or phenotyping, and a failure to look for associations with specific subtypes of stroke [15]. Meta-analysis of published candidate gene studies has revealed some consistently positive findings however, such as Factor V Leiden Arg506Gln, MTHFR C677T and the ACE insertion/deletion polymorphism [16], although caution is required in interpretation due to the possible effect of publication bias meaning positive studies are more likely to be published. Although still useful when explaining specific hypotheses, candidate gene studies have now been largely superseded by the genome-wide association study (GWAS) technique.

Familial linkage studies examine genetic variants through multiple generations of families and correlate these with disease incidence. Associations with a specific gene are not sought using this approach, but rather one looks for variants anywhere in the entire genome, and they are therefore referred to as “nonhypothesis driven” experiments. The technique has had, and continues to have, great success in identifying genes underlying Mendelian disorders in monogenic conditions where a single gene contributes the entirety of genetic risk. Familial linkage studies rely on collection of families with the disease however, and this is a challenge in stroke where the late age of onset means parents are often not alive; this has hampered collection of cases in studies such as the siblings with ischaemic stroke study (SWISS) [17, 18]. 

 One notable exception to this has been in Iceland, where the DeCode group reported identification of the first genetic risk for common polygenic ischaemic stroke via such a familial linkage study, which they named *STRK1* [19]. This study used the unique national collection of genealogical samples and family structures tracked in the Icelandic population to retrospectively determine cause of death and provide material for genotyping. The *STRK1* locus was identified as overlying the gene phosphodiesterase4D (PDE4D), a cyclic AMP regulator which is a plausible biological candidate [19]. Subsequent replication in European cohorts failed to confirm these findings [20]. This study was undertaken as large-scale genome-wide experiments were being developed as a mainstream technique. By current standards the DeCode finding would today be considered underpowered as it failed to exceed the currently agreed statistical threshold for such studies.

## 3. Identification of Genetic Risk: The Genome Wide Association Study

The field of complex genetics has been revolutionized by the advent of the genome-wide association study (GWAS) [21]. This can be thought of as a large series of candidate gene studies performed in a single experiment on an array based format. As many as 1.2 million polymorphisms at a time can now be studied in this manner. Crucially, these are spread throughout the entire genome and such experiments are thus nonhypothesis driven, overcoming a major limitation of the candidate gene study. Such a large number of experiments in a single study requires a large sample sizes to allow sufficient power, even after statistical correction for multiple comparisons. Also crucial to progress has been the realisation that careful phenotyping is important, and that associations should be replicated in a second population before publication. 

An early demonstration of the power of this technique was in age-related macular degeneration, a late onset eye disorder leading to blindness in which conventional cardiovascular risk factors play a part. Applying a GWAS approach to this condition revealed associations with the complement factor H gene, and identification of a single amino acid substitution which proved to be the causal variant in this condition [22]. Interestingly, the same locus had been identified by a familial linkage approach in previous studies, but refinement of the region and identification of the causal variant via familial linkage had been impossible. 

As a consequence of this and other studies, the enormous potential of GWAS to identify common variants associated with common diseases became recognised, with perhaps the seminal GWAS publication by the Wellcome Trust Case Control Consortium 1 study making GWAS a mainstream technique in disease gene identification [23]. This study examined 14,000 cases of seven common diseases and 3,000 shared controls in an effort to identify genetic variants in human disease. Investigating bipolar disorder, coronary artery disease, Crohn's disease, hypertension, rheumatoid arthritis, and type I and type II diabetes, this single study identified over 58 novel loci as potentially contributing genetic risk in these conditions. To date, the GWAS technique has identified over 1212 new genetic loci predisposing to common polygenic disease (http://www.genome.gov/gwastudies). Novel genetic associations with a range of cardiovascular phenotypes including myocardial infarction, coronary artery disease, diabetes and hyperlipidaemia have been reported, but few variants have been confirmed for ischaemic stroke. 

It should be noted that while GWAS is a powerful technique, it requires very large, well phenotyped case series—typically in the thousands of samples, and even with these sample sizes is powered only to detect modest risks, typically with odds ratios in the region of 1.2–1.5. Thus the contribution of each risk locus to overall disease incidence is likely to be minor, although these risks are additive and as such identification of multiple loci may allow individual risk profiles to be determined. Identification of high risk individuals could be useful in early intervention to reduce conventional risk factors, more rigorous screening for early signs of disease and in investigating severity of disease at onset as well as associations with disease recurrence.

## 4. GWAS and Ischaemic Stroke

While GWAS has contributed greatly to identification of genetics of many complex diseases over the last 5 years, application of the technique to ischaemic stroke has been slower, with large-scale collaborative efforts only now beginning to emerge. An early study applied the GWAS technique to 249 ischaemic stroke cases and 268 controls, but we now realize this was underpowered [24]. A more recent study in prospective population-based cohorts identified a region on Chromosome 12 overlying the NINJ2 gene in ischaemic stroke cases [25], although a subsequent large replication failed to confirm this finding [26].

The collection of large, well phenotyped sample cohorts for genetic analysis in stroke presents major challenges. In particular phenotyping, which we now realize is essential, requires detailed and expensive investigations. As in other complex diseases, collection of sufficiently large sample sizes depends on larges scale international collaborations, and to address this the International Stroke Genetics Consortium (ISGC—http://www.strokegentics.org/) was established. Currently an ischaemic stroke GWAS in 4000 cases is near completion as part of the Wellcome Trust Case Control Consortium 2 study (WTCCC2) in collaboration with the ISGC. GWAS studies in countries including the US and Australia are also ongoing with results expected in 2011. A lesson from other disease areas is that, even with sample sizes of several thousands, power is limited and meta-analysis of multiple GWAS studies has become standard practice. The Meta-stroke collaboration has been formed in ischaemic stroke to address this. 

These collaborative efforts have achieved early success in ischaemic stroke via examination of genetic associations already identified in related cardiovascular diseases. The identification of a region on Chromosome 9p21 in myocardial infarction and coronary artery disease, which surrounds the CDKN2A and CDKN2B genes, has generated a large amount of interest [27]. An examination of this locus in a candidate gene study in ischaemic stroke cases revealed an association with large artery stroke, but not the other ischaemic stroke subtypes [28]. This association persisted across multiple populations and importantly emphasises the likely differing contributions of genetic risks to different ischaemic stroke subtypes. 

 Two genetic variants identified as contributing to the risk of atrial fibrillation (AF), in the genes PITX2 and ZFHX3, have also been shown to associate with cardioembolic stroke risk for which AF is an important risk factor [29, 30]. As new loci are identified for other cardiovascular diseases which themselves are associated with stroke, rapid testing these in stroke populations via large International collaborations is possible.

## 5. Recommendations for Future Genetic Studies in Stroke

Previous studies in stroke genetics have been disappointing. There are a number of reasons for this, most significant of which are poor phenotyping, small sample sizes, and failure to replicate initial findings in a second population. Any future genetic study, whether hypothesis driven or nonhypothesis driven, should address each of these issues prior to publication. Power calculations demonstrating the number of cases required for confirmation or refutation of a finding should be included to allow an estimate of the significance and robustness of the findings presented. Genetic risks in stroke are usually estimated to be between 1.1 and 1.5 for a single loci, and studies should be adequately powered (i.e., be comprised of sufficient cases) to detect risks of this size. Replication of positive associations prior to publication is important. This should be in a separate case series using a different control set. 

Increasing evidence suggests genetic risks differ depending on ischaemic stroke subtype. Future genetic studies should therefore include reference to subtypes and subtype specific risks. Evidence of genetic risk in a homogenous population of ischaemic stroke without subtype investigation is likely to lead to spurious associations.

While these measures lead to increased cost and complexity of studies, it is only through such robust experimental procedures that we will truly begin to understand the genetic risks of stroke and how these are manifest.

## 6. The Post GWAS Era in Stroke Genetics

Genome-wide association studies have been specifically conceived to address the common variant, common disease (CVCD) hypothesis. This concept underlies the majority of genetic studies to date not just in stroke but in other common diseases. According to the CVCD hypothesis, multiple genetic risk factors contribute to disease, each with a small additional increase in risk. These risks are additive in nature and together provide an individual risk profile that allows for a significant genetic contribution. In order for this hypothesis to hold true however, variants have to be common in the population.

Despite the success of GWAS in identifying susceptibility loci, for the vast majority of diseases these account for only a fraction of the heritability initially attributed to genetic risk factors. Each risk identified carries a much smaller risk than originally thought under the CVCD hypothesis. For this reason the CVCD theory of genetic risk is now being questioned, and various mechanisms have been suggested to account for this “missing heritability” [31]. An alternative hypothesis is that rare variants are important in common disease risk (RVCD). This states that as well as common variants that each contribute very small risks, susceptible individuals may carry variants of higher risk which are rare and perhaps even private to themselves or closely related family members. Such risks would not be detectable via classical familial linkage since there would be multiple risk variants contributing to an individuals susceptibility to disease, but neither would they be detectable via GWAS since they would be specific to individuals or closely related family members and therefore not carried by the rest of the population. Under this hypothesis any one individual would be expected to carry many variants detectable by GWAS, and a handful of higher risk alleles in a private manner. Together these combine to produce an individuals overall risk profile of disease susceptibility, and may account for the so called “hidden heritability” conundrum which persists after GWAS. Identification of these rare variants requires a sequencing approach which provides information on every base pair across the region of interest, and this approach is provided by next generation sequencing (NGS). 

 NGS has arisen from both advances in technology, and from our ability to sequence the human genome as a consequence of the human genome project (http://www.hapmap.org/) and the 1000 genome (http://www.1000genomes.org/) project among others. It is now possible to obtain the entire coding sequence of the human genome in less than a week, and to make comparisons between genomes due to advances in computational methods and processing power. Sequencing of targeted regions at a previously unparalleled depth and fold coverage without the need for generation of vector libraries or bacterial culture is now routine and provided by a number of service providers using a variety of techniques [32]. While currently expensive, such techniques give access to perhaps the majority of the information that conventional genetics can be expected to provide, namely the entire coding sequence of the genome. Interpretation of that sequence is still in its relative infancy.

## 7. Beyond Genomics in Identifying Ischaemic Stroke Risk Factors

Understanding the genetic basis of disease risk requires an understanding of the way in which these genes have their effects in the body. Genes code for RNA, which is then translated into protein. These proteins can act alone, in multiples of themselves as homodimers or in conjunction with other proteins to form heterodimers. Similarly genes may interact with each other, so called epigenetics, or may interact with environmental factors in gene-environment interactions which only affect disease risk when both the environmental and genetic components of the interaction are present. Detecting such gene-gene or gene-environment interactions requires much larger sample sizes. Their importance has been shown in other cardiovascular diseases [33] and in association studies with the quantitative trait continuous carotid IMT [34]. 

 Examination of RNA and proteins in a nonhypothesis driven manner, similar to GWAS for DNA, is also possible. Examination of the transcriptome is a relatively old technique via the use of microarrays, and it is this technology which actually led to the development of GWAS using DNA slides or cartridges rather than RNA and cDNA-based ones. More recently studies have been examining the possibility of ignoring the DNA level and trying to perform transcription profiling (examination of all the RNA's being produced at a specific time point). By examining changes in the level of transcription of a subset of RNAs and correlating these with changes in disease state, disease subtype or disease severity, we may be able to better understand how genetic differences influence disease processes [35]. 

 There are reports that it is possible to differentiate between different stroke subtypes using this methodology, with expression levels of just 23 genes being able to differentiate between cardioembolic stroke and large vessel disease [36]. While such studies are not yet able to replace conventional investigative techniques for determining ischaemic stroke subtype, identification of a expression profiles may give novel insights into stroke pathogenesis, and perhaps identification of suitable biomarkers for monitoring risk reduction treatments. This technique has also been applied to associated phenotypes such as white matter hyperintensity in the brain, currently only detectable by MRI [37].

## 8. Conclusions

Considerable evidence suggests genetic factors are important in ischaemic stroke risk. The advent of new techniques such as GWAS has contributed enormously to the understanding of the genetics of other complex disease and progress is just beginning to be made in stroke. For success large, well phenotyped case cohorts are required, and international collaborations are essential. NGS technology and techniques such as transcription profiling and proteomics will allow us to look for rarer variants in stroke cases and attempt to identify how these exert their effects at the molecular level, but whether these will be important remains to be determined. 

## Figures and Tables

**Box 1:**
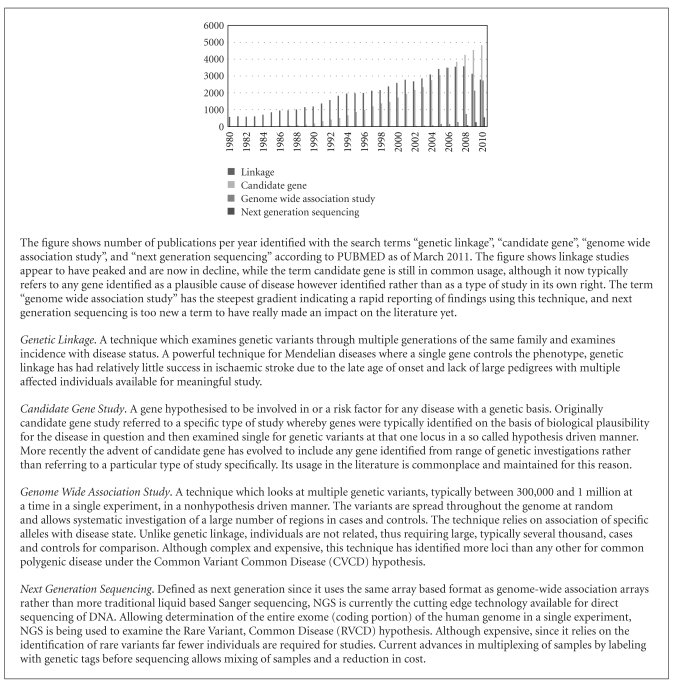
Types of analysis for genetic investigation.

## References

[1] Floßmann E, Schulz UGR, Rothwell PM (2004). Systematic review of methods and results of studies of the genetic epidemiology of ischemic stroke. *Stroke*.

[2] Jerrard-Dunne P, Cloud G, Hassan A, Markus HS (2003). Evaluating the genetic component of ischemic stroke subtypes: a family history study. *Stroke*.

[3] Polychronopoulos P, Gioldasis G, Ellul J (2002). Family history of stroke in stroke types and subtypes. *Journal of the Neurological Sciences*.

[4] Rubattu S, Volpe M, Kreutz R, Ganten U, Ganten D, Lindpaintner K (1996). Chromosomal mapping of quantitative trait loci contributing to stroke in a rat model of complex human disease. *Nature Genetics*.

[5] Duggirala R, Villalpando CG, O’Leary DH, Stern MP, Blangero J (1996). Genetic basis of variation in carotid artery wall thickness. *Stroke*.

[6] Jartti L, Ronnemaa T, Kaprio J (2002). Population-based twin study of the effects of migration from Finland to Sweeden on endothelial function and intima-media thickness. *Arteriosclerosis, Thrombosis, and Vascular Biology*.

[7] Moskau S, Golla A, Grothe C, Boes M, Pohl C, Klockgether T (2005). Heritability of carotid artery atherosclerotic lesions: an ultrasound study in 154 families. *Stroke*.

[8] Carmelli D, DeCarli C, Swan GE (1998). Evidence for genetic variance in white matter hyperintensity volume in normal elderly male twins. *Stroke*.

[9] Atwood LD, Wolf PA, Heard-Costa NL (2004). Genetic variation in white matter hyperintensity volume in the Framingham study. *Stroke*.

[10] Turner ST, Fornage M, Jack CR (2009). Genomic susceptibility loci for brain atrophy, ventricular volume, and leukoaraiosis in hypertensive sibships. *Archives of Neurology*.

[11] Sinner MF, Ellinor PT, Meitinger T, Benjamin EJ, Kääb S (2011). Genome-wide association studies of atrial fibrillation: past, present, and future. *Cardiovascular Research*.

[12] Helgadottir A, Thorleifsson G, Magnusson KP (2008). The same sequence variant on 9p21 associates with myocardial infarction, abdominal aortic aneurysm and intracranial aneurysm. *Nature Genetics*.

[13] Hassan A, Markus HS (2000). Genetics and ischaemic stroke. *Brain*.

[14] Tonk M, Haan J (2007). A review of genetic causes of ischemic and hemorrhagic stroke. *Journal of the Neurological Sciences*.

[15] Dichgans M, Markus HS (2005). Genetic association studies in stroke: methodological issues and proposed standard criteria. *Stroke*.

[16] Casas JP, Hingorani AD, Bautista LS, Sharma P (2004). Meta-analysis of genetic studies in ischaemic stroke: thirty two genes involving approximately 18000 cases and 58000 controls. *Archives of Neurology*.

[17] Meschia JF, Brown RD, Brott TG, Hardy J, Atkinson EJ, O’Brien PC (2001). Feasibility of an affected sibling pair study in ischemic stroke: results of a 2-center family history registry. *Stroke*.

[18] Worrall BB, Chen DT, Brown RD, Brott TG, Meschia JF (2005). A survey of the SWISS researchers on the impact of sibling privacy protections on pedigree recruitment. *Neuroepidemiology*.

[19] Gretarsdottir S, Thorleifsson G, Reynisdottir ST (2003). The gene encoding phosphodiesterase 4D confers risk of ischemic stroke. *Nature Genetics*.

[20] Bevan S, Dichgans M, Gschwendtner A, Kuhlenbäumer G, Ringelstein EB, Markus HS (2008). Variation in the PDE4D gene and ischemic stroke risk: a systematic review and meta-analysis on 5200 cases and 6600 controls. *Stroke*.

[21] Hardy J, Singleton A (2009). Genomewide association studies and human disease. *The New England Journal of Medicine*.

[22] Edwards AO, Ritter R, Abel KJ, Manning A, Panhuysen C, Farrer LA (2005). Complement factor H polymorphism and age-related macular degeneration. *Science*.

[23] The Wellcome Trust Case Control Consortium (2007). Genome-wide association study of 14,000 cases of seven common diseases and 3,000 shared controls. *Nature*.

[24] Matarín M, Brown WM, Scholz S (2007). A genome-wide genotyping study in patients with ischaemic stroke: initial analysis and data release. *The Lancet Neurology*.

[25] Ikram MA, Seshadri S, Bis JC (2009). Genomewide association studies of stroke. *The New England Journal of Medicine*.

[26] International Stroke Genetic Consortium (2010). Failure to validate association between 12p13 variants and ischaemic stroke. *The New England Journal of Medicine*.

[27] Schunkert H, Götz A, Braund P (2008). Repeated replication and a prospective meta-analysis of the association between chromosome 9p21.3 and coronary artery disease. *Circulation*.

[28] Gschwendtner A, Bevan S, Cole JW (2009). Sequence variants on chromosome 9p21.3 confer risk for atherosclerotic stroke. *Annals of Neurology*.

[29] Gudbjartsson DF, Arnar DO, Helgadottir A (2007). Variants conferring risk of atrial fibrillation on chromosome 4q25. *Nature*.

[30] Gretarsdottir S, Thorleifsson G, Manolescu A (2008). Risak variants for atrial fibrillation on chromosome 4q25 associate with ischaemic stroke. *Annals of Neurology*.

[31] Manolio TA, Collins FS, Cox NJ (2009). Finding the missing heritability of complex diseases. *Nature*.

[32] Metzker ML (2010). Sequencing technologies—the next generation. *Nature Reviews Genetics*.

[33] Talmud PJ, Humphries SE (2004). Gene: environment interactions and coronary heart disease risk. *World Review of Nutrition and Dietetics*.

[34] Markus HS, Labrum R, Bevan S (2006). Genetic and acquired inflammatory conditions are synergistically associated with early carotid atherosclerosis. *Stroke*.

[35] Stamova B, Xu H, Jickling G (2010). Gene expression profiling of blood for the prediction of ischemic stroke. *Stroke*.

[36] Xu H, Tang Y, Liu DZ (2008). Gene expression in peripheral blood differs after cardioembolic compared with large-vessel atherosclerotic stroke: biomarkers for the etiology of ischemic stroke. *Journal of Cerebral Blood Flow and Metabolism*.

[37] Xu H, Stamova B, Jickling G (2010). Distinctive RNA expression profiles in blood associated with white matter hyperintensities in brain. *Stroke*.

